# (3,5-Dinitro-1,3,5-triazinan-1-yl)methanone

**DOI:** 10.1107/S1600536809041531

**Published:** 2009-10-17

**Authors:** Qiao-Ling Zhang, Xiao-Feng Qu, Jian-Long Wang

**Affiliations:** a School of Chemical Engineering and Environment, North University of China, Taiyuan, People’s Republic of China

## Abstract

In the title compound, C_5_H_9_N_5_O_5_, prepared from hexa­mine by acetyl­ation and nitration, the triazine ring adopts a chair conformation with all three substituent groups lying on the same side of the ring.

## Related literature

For the Bachmann process, see: Bachmann & Sheehan (1949 ▶). For the synthesis, see: Warman *et al.* (1973 ▶). For a related structure, see: Choi *et al.* (1975 ▶).
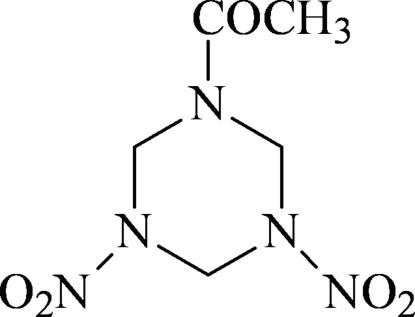


## Experimental

### 

#### Crystal data

C_5_H_9_N_5_O_5_
*M* *_r_* = 219.17
Monoclinic, 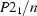

*a* = 8.8972 (18) Å
*b* = 10.061 (2) Å
*c* = 9.890 (2) Å
β = 100.42 (3)°
*V* = 870.7 (3) Å^3^
*Z* = 4
Mo *K*α radiation
μ = 0.15 mm^−1^
*T* = 293 K
0.50 × 0.50 × 0.40 mm

#### Data collection

Rigaku R-AXIS RAPID IP diffractometer
Absorption correction: multi-scan (**ABSCOR**; Higashi, 1995 ▶) *T*_min_ = 0.929, *T*_max_ = 0.943
3599 measured reflections
1988 independent reflections
1419 reflections with *I* > 2σ(*I*)
*R*_int_ = 0.028

#### Refinement

*R*[*F* ^2^ > 2σ(*F* ^2^)] = 0.051
*wR*(*F* ^2^) = 0.142
*S* = 1.03
1988 reflections
138 parameters
H-atom parameters constrained
Δρ_max_ = 0.28 e Å^−3^
Δρ_min_ = −0.26 e Å^−3^

### 

Data collection: *RAPID-AUTO* (Rigaku, 2000 ▶); cell refinement: *RAPID-AUTO*; data reduction: *CrystalStructure* (Rigaku/MSC, 2000 ▶); program(s) used to solve structure: *SHELXS97* (Sheldrick, 2008 ▶); program(s) used to refine structure: *SHELXL97* (Sheldrick, 2008 ▶); molecular graphics: *SHELXTL* (Sheldrick, 2008 ▶); software used to prepare material for publication: *SHELXL97*.

## Supplementary Material

Crystal structure: contains datablocks I, global. DOI: 10.1107/S1600536809041531/zs2012sup1.cif

Structure factors: contains datablocks I. DOI: 10.1107/S1600536809041531/zs2012Isup2.hkl

Additional supplementary materials:  crystallographic information; 3D view; checkCIF report

**Fig. 1.**
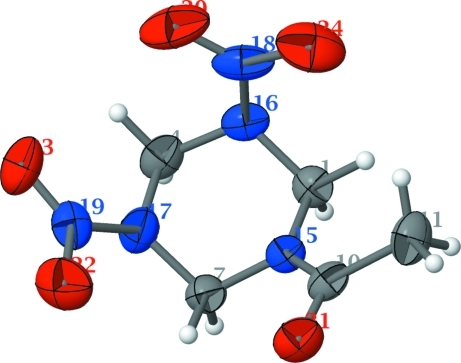
The molecular structure and atom numbering scheme for the title compound (I). Non-H atoms are shown as 50% probability displacement ellipsoids.

Enhanced figure: interactive version of Fig. 1

**Fig. 2.**
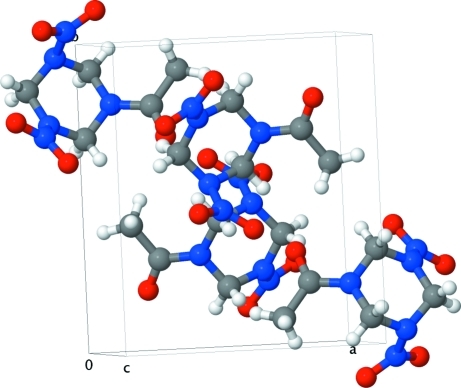
The packing of the title compound, viewed down the c axis of the unit cell.

Enhanced figure: interactive version of Fig. 2

## supplementary crystallographic information

### Comment

1-Acetyl-3,5-dinitro-1,3,5-triazinane (1-acetylhexahydro-3,5-dinitro-1,3,5-triazine) (I) is obtained as a by-product in the synthesis of hexahydro-1,3,5-trinitro-1,3,5-triazine (RDX) from 1,3,5,7 -tetraazaadamantane (hexamine) by the Bachmann process (Bachmann & Sheehan, 1949). As part of our search for the reaction mechanism involved in the nitrolysis of hexamine, we synthesized the title compound, and describe its structure here.

In (I), the hexahydrotriazine ring adopts a chair conformation with all three substituent groups lying on the same side of the triazine ring.The ring bond distances and angles are almost identical (the maximum deviation from the average C—N bond distance [1.44 (8) Å] is 0.01Å and the maximum deviation from the average bond angle [112 (3)°] is 3°). The three ring N atoms are equally distant from the plane through the C atoms (C1, C2 and C3) (0.40±0.06 (2) Å). This deviation is slightly larger than that found in hexahydro-1,3,5-triacetyl-1,3,5-triazine (TRAT) (Choi *et al.*, 1975), due to the three different substituent groups on the hexahydrotriazine ring in (I), whereas in TRAT, the three groups are the same. In (I) the three substituent groups are essentially planar with maximum deviations from the mean plane of these groups for atoms N1, N2 and N3 of 0.02 (4), 0.09 (6) and 0.10 (3) Å respectively.

### Experimental

The title compound was prepared from hexamine according to a literature method (Warman *et al.*, 1973). Crystals suitable for X-ray analysis were obtained by slow evaporation of an nitromethane solution at room temperature.

### Refinement

All H atoms were positioned geometrically and treated as riding, with C—H bond lengths constrained to 0.97 Å (alicyclic CH), 0.96 Å (methyl CH), and with*U*_iso_(H) = 1.2 (1.5 for methyl groups) times *U*_eq_(C).

### Crystal data

**Table:**

C_5_H_9_N_5_O_5_	*F*(000) = 456
*M**_r_* = 219.17	*D*_x_ = 1.672 Mg m^−^^3^
Monoclinic, *P*2_1_/*n*	Melting point: 431(2) K
Hall symbol: -P 2yn	Mo *K*α radiation, λ = 0.71073 Å
*a* = 8.8972 (18) Å	Cell parameters from 3599 reflections
*b* = 10.061 (2) Å	θ = 2.8–27.5°
*c* = 9.890 (2) Å	µ = 0.15 mm^−^^1^
β = 100.42 (3)°	*T* = 293 K
*V* = 870.7 (3) Å^3^	Block, colorless
*Z* = 4	0.50 × 0.50 × 0.40 mm

### Data collection

**Table:**

Rigaku R-AXIS RAPID IP diffractometer	1988 independent reflections
Radiation source: fine-focus sealed tube	1419 reflections with *I* > 2σ(*I*)
graphite	*R*_int_ = 0.028
Detector resolution: 10.00 pixels mm^-1^	θ_max_ = 27.5°, θ_min_ = 2.8°
ω scans	*h* = −11→11
Absorption correction: multi-scan (*ABSCOR*; Higashi, 1995)	*k* = −13→13
*T*_min_ = 0.929, *T*_max_ = 0.943	*l* = −12→12
3599 measured reflections	

### Refinement

**Table:**

Refinement on *F*^2^	Secondary atom site location: difference Fourier map
Least-squares matrix: full	Hydrogen site location: inferred from neighbouring sites
*R*[*F*^2^ > 2σ(*F*^2^)] = 0.051	H-atom parameters constrained
*wR*(*F*^2^) = 0.142	*w* = 1/[σ^2^(*F*_o_^2^) + (0.09*P*)^2^] where *P* = (*F*_o_^2^ + 2*F*_c_^2^)/3
*S* = 1.03	(Δ/σ)_max_ = 0.002
1988 reflections	Δρ_max_ = 0.28 e Å^−^^3^
138 parameters	Δρ_min_ = −0.26 e Å^−^^3^
0 restraints	Extinction correction: *SHELXL97* (Sheldrick, 2008), Fc^*^=kFc[1+0.001xFc^2^λ^3^/sin(2θ)]^-1/4^
Primary atom site location: structure-invariant direct methods	Extinction coefficient: 0.242 (16)

### Special details

**Table:**

Geometry. All e.s.d.'s (except the e.s.d. in the dihedral angle between two l.s. planes) are estimated using the full covariance matrix. The cell e.s.d.'s are taken into account individually in the estimation of e.s.d.'s in distances, angles and torsion angles; correlations between e.s.d.'s in cell parameters are only used when they are defined by crystal symmetry. An approximate (isotropic) treatment of cell e.s.d.'s is used for estimating e.s.d.'s involving l.s. planes.

### Fractional atomic coordinates and isotropic or equivalent isotropic displacement parameters (Å^2^)

**Table:**

	*x*	*y*	*z*	*U*_iso_*/*U*_eq_	
C1	0.47469 (19)	0.41301 (16)	0.32195 (16)	0.0389 (4)	
H1A	0.5005	0.3837	0.4168	0.047*	
H1B	0.4000	0.4839	0.3170	0.047*	
C2	0.72467 (19)	0.35894 (19)	0.26987 (18)	0.0456 (4)	
H2A	0.8044	0.3941	0.2248	0.055*	
H2B	0.7713	0.3329	0.3624	0.055*	
C3	0.51659 (19)	0.19589 (16)	0.23350 (19)	0.0429 (4)	
H3A	0.4708	0.1292	0.1680	0.052*	
H3B	0.5392	0.1547	0.3236	0.052*	
C4	0.26587 (18)	0.29469 (17)	0.16314 (15)	0.0385 (4)	
C5	0.1604 (2)	0.4079 (2)	0.1715 (2)	0.0531 (5)	
H5A	0.0627	0.3892	0.1158	0.080*	
H5B	0.2020	0.4873	0.1390	0.080*	
H5C	0.1485	0.4201	0.2653	0.080*	
N1	0.41055 (15)	0.30361 (13)	0.23592 (13)	0.0357 (3)	
N2	0.61111 (16)	0.46206 (14)	0.27642 (13)	0.0405 (4)	
N3	0.65682 (16)	0.24375 (17)	0.19558 (16)	0.0491 (4)	
N4	0.5835 (2)	0.54679 (16)	0.16262 (16)	0.0550 (5)	
N5	0.69287 (18)	0.21008 (15)	0.07159 (15)	0.0468 (4)	
O1	0.6820 (2)	0.55437 (17)	0.09159 (16)	0.0839 (6)	
O2	0.22571 (14)	0.19538 (13)	0.09572 (13)	0.0525 (4)	
O3	0.6163 (2)	0.12455 (17)	0.00671 (15)	0.0723 (5)	
O4	0.80447 (17)	0.26203 (18)	0.03929 (15)	0.0685 (5)	
O5	0.4676 (2)	0.61206 (16)	0.14783 (19)	0.0819 (6)	

### Atomic displacement parameters (Å^2^)

**Table:**

	*U*^11^	*U*^22^	*U*^33^	*U*^12^	*U*^13^	*U*^23^
C1	0.0417 (9)	0.0443 (9)	0.0292 (7)	−0.0021 (7)	0.0023 (6)	−0.0039 (6)
C2	0.0363 (9)	0.0593 (11)	0.0390 (9)	−0.0075 (8)	0.0013 (7)	−0.0027 (8)
C3	0.0415 (9)	0.0399 (9)	0.0479 (9)	−0.0033 (7)	0.0094 (7)	0.0018 (7)
C4	0.0371 (8)	0.0507 (9)	0.0274 (7)	−0.0073 (7)	0.0055 (6)	0.0032 (7)
C5	0.0363 (9)	0.0751 (13)	0.0465 (10)	0.0059 (9)	0.0041 (7)	0.0011 (9)
N1	0.0341 (7)	0.0390 (7)	0.0327 (7)	−0.0025 (5)	0.0024 (5)	−0.0012 (5)
N2	0.0441 (8)	0.0439 (8)	0.0297 (7)	−0.0106 (6)	−0.0034 (6)	0.0009 (6)
N3	0.0420 (8)	0.0595 (9)	0.0480 (9)	−0.0067 (7)	0.0141 (6)	−0.0122 (7)
N4	0.0761 (11)	0.0453 (9)	0.0368 (8)	−0.0260 (8)	−0.0076 (8)	0.0032 (7)
N5	0.0487 (9)	0.0542 (9)	0.0368 (8)	0.0130 (7)	0.0059 (7)	0.0037 (7)
O1	0.1182 (14)	0.0832 (12)	0.0533 (9)	−0.0413 (11)	0.0236 (10)	0.0118 (8)
O2	0.0479 (7)	0.0615 (8)	0.0450 (7)	−0.0159 (6)	0.0006 (6)	−0.0099 (6)
O3	0.1003 (13)	0.0668 (9)	0.0496 (8)	−0.0102 (9)	0.0129 (8)	−0.0185 (7)
O4	0.0577 (9)	0.0945 (11)	0.0598 (9)	0.0010 (9)	0.0281 (7)	−0.0005 (8)
O5	0.0946 (13)	0.0625 (10)	0.0768 (12)	0.0044 (9)	−0.0163 (9)	0.0260 (9)

### Geometric parameters (Å, °)

**Table:**

C1—N1	1.4440 (19)	C4—O2	1.2184 (19)
C1—N2	1.455 (2)	C4—N1	1.359 (2)
C1—H1A	0.9700	C4—C5	1.487 (2)
C1—H1B	0.9700	C5—H5A	0.9600
C2—N3	1.445 (2)	C5—H5B	0.9600
C2—N2	1.458 (2)	C5—H5C	0.9600
C2—H2A	0.9700	N2—N4	1.398 (2)
C2—H2B	0.9700	N3—N5	1.365 (2)
C3—N1	1.440 (2)	N4—O5	1.209 (2)
C3—N3	1.449 (2)	N4—O1	1.220 (2)
C3—H3A	0.9700	N5—O3	1.208 (2)
C3—H3B	0.9700	N5—O4	1.215 (2)
			
N1—C1—N2	109.82 (13)	C4—C5—H5A	109.5
N1—C1—H1A	109.7	C4—C5—H5B	109.5
N2—C1—H1A	109.7	H5A—C5—H5B	109.5
N1—C1—H1B	109.7	C4—C5—H5C	109.5
N2—C1—H1B	109.7	H5A—C5—H5C	109.5
H1A—C1—H1B	108.2	H5B—C5—H5C	109.5
N3—C2—N2	111.37 (13)	C4—N1—C3	120.14 (13)
N3—C2—H2A	109.4	C4—N1—C1	126.71 (14)
N2—C2—H2A	109.4	C3—N1—C1	113.15 (13)
N3—C2—H2B	109.4	N4—N2—C1	114.91 (14)
N2—C2—H2B	109.4	N4—N2—C2	114.86 (15)
H2A—C2—H2B	108.0	C1—N2—C2	113.33 (13)
N1—C3—N3	110.59 (14)	N5—N3—C2	120.76 (15)
N1—C3—H3A	109.5	N5—N3—C3	120.23 (15)
N3—C3—H3A	109.5	C2—N3—C3	115.80 (14)
N1—C3—H3B	109.5	O5—N4—O1	125.60 (18)
N3—C3—H3B	109.5	O5—N4—N2	116.75 (17)
H3A—C3—H3B	108.1	O1—N4—N2	117.51 (19)
O2—C4—N1	119.99 (16)	O3—N5—O4	125.20 (17)
O2—C4—C5	122.20 (16)	O3—N5—N3	116.89 (16)
N1—C4—C5	117.81 (15)	O4—N5—N3	117.74 (16)
			
O2—C4—N1—C3	0.0 (2)	N2—C2—N3—N5	112.60 (17)
C5—C4—N1—C3	178.88 (14)	N2—C2—N3—C3	−47.2 (2)
O2—C4—N1—C1	−179.01 (15)	N1—C3—N3—N5	−110.68 (18)
C5—C4—N1—C1	−0.2 (2)	N1—C3—N3—C2	49.2 (2)
N3—C3—N1—C4	126.86 (15)	C1—N2—N4—O5	28.9 (2)
N3—C3—N1—C1	−53.98 (18)	C2—N2—N4—O5	163.00 (15)
N2—C1—N1—C4	−123.74 (16)	C1—N2—N4—O1	−155.26 (16)
N2—C1—N1—C3	57.17 (17)	C2—N2—N4—O1	−21.1 (2)
N1—C1—N2—N4	80.18 (16)	C2—N3—N5—O3	−169.79 (16)
N1—C1—N2—C2	−54.65 (17)	C3—N3—N5—O3	−10.9 (2)
N3—C2—N2—N4	−85.27 (16)	C2—N3—N5—O4	14.7 (2)
N3—C2—N2—C1	49.60 (18)	C3—N3—N5—O4	173.59 (16)
